# Gene model for the ortholog of *ImpL2* in *Drosophila simulans*

**DOI:** 10.17912/micropub.biology.000715

**Published:** 2024-11-30

**Authors:** Megan E. Lawson, Graham M. Jones, Megan Runion, Drew Sims, Austin Young, Olivia Briggs, Lindsey J. Long, Solomon Tin Chi Chak, Indrani Bose, Chinmay P. Rele, Laura K Reed

**Affiliations:** 1 The University of Alabama, Tuscaloosa, AL USA; 2 Oklahoma Christian University, Edmond, OK USA; 3 SUNY Old Westbury, Old Westbury, NY USA; 4 Western Carolina University, Cullowhee, NC USA; 5 Biological Sciences, The University of Alabama, Tuscaloosa, AL 35487

## Abstract

Gene model for the ortholog of *Ecdysone-inducible gene L2* ( *ImpL2* ) in the May 2017 (Princeton ASM75419v2/DsimGB2) Genome Assembly (GenBank Accession: GCA_000754195.3 ) of *Drosophila simulans* . This ortholog was characterized as part of a developing dataset to study the evolution of the Insulin/insulin-like growth factor signaling pathway (IIS) across the genus *Drosophila* using the Genomics Education Partnership gene annotation protocol for Course-based Undergraduate Research Experiences.

**Figure 1. Genomic neighborhood and gene model for ImpL2 in D. simulans:**
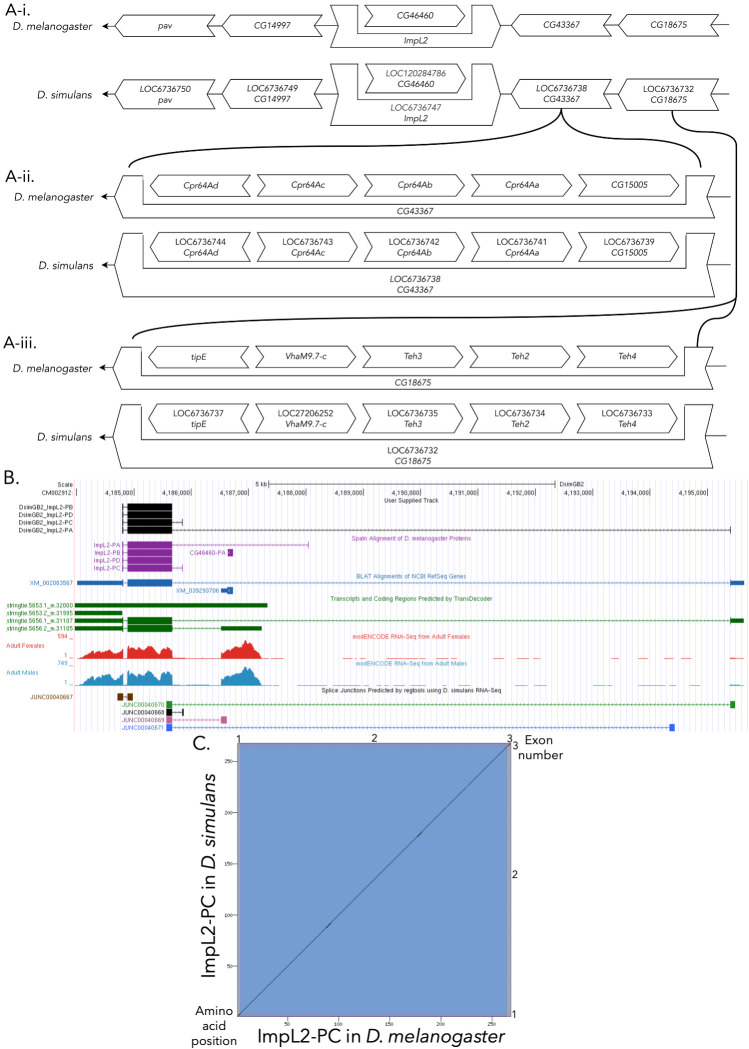
(A-i) **Synteny of genomic neighborhood of *ImpL2* in both *D. melanogaster* and *D. simulans* .** Gene arrows pointing in the same direction as target gene in both *D. simulans* and *D. melanogaster* are on the same strand as the target gene; gene arrows pointing in the opposite direction are on the opposite strand. The thin underlying arrows pointing to the left indicate that *ImpL2* is on the – strands. White arrows in *D. simulans* indicate the locus ID and the orthology to the corresponding gene in *D. melanogaster* . The gene names given in the *D. simulans* gene arrows indicate the orthologous gene in *D. melanogaster* , while the locus identifiers are specific to *D. simulans* . (A-ii) Nested genes within *CG43367 ,* shown in the same format as figure A-i. (A-iii) Nested genes within *CG18675 ,* shown in the same format as figure A-i *.* (B) **Gene Model in UCSC Track Hub** (Raney et al. 2014): Image from the Track Hub instance housing the gene model in *D. simulans* , Spaln of *D. melanogaster* Proteins (alignment of Refseq proteins from *D. melanogaster* ), BLAT alignments of NCBI RefSeq Genes (alignment of Refseq genes for *D. simulans* ), RNA-Seq from adult male and females (alignment of Illumina RNA-Seq reads from *D. simulans* ), and Transcripts and Coding Regions Predicted by TransDecoder and Splice Junctions Predicted by regtools using *D. simulans* RNA-Seq (Gravely et al. 2011; SRP006203 ). Splice junctions shown have a minimum read-depth of 9 with 1-9, 10-49, 50-99, 100-499, 500-999 supporting reads in black, blue, green, pink, and brown, respectively. The custom gene model (User Supplied Track) is indicated in black with CDSs depicted with boxes and introns with narrow lines (arrows indicate direction of transcription). (C) **Dot Plot of ImpL2-PC in *D. melanogaster* ( *x* -axis) vs. the orthologous peptide in *D. simulans* ( *y* -axis)** . Amino acid number is indicated along the left and bottom; CDS number is indicated along the top and right, and CDSs are also highlighted with alternating colors. Note that the first and last CDSs of Impl2 are very small, so their corresponding bands of color are very thin.

## Description

**Table:** 

* This article reports a predicted gene model generated by undergraduate work using a structured gene model annotation protocol defined by the Genomics Education Partnership (GEP; thegep.org ) for Course-based Undergraduate Research Experience (CURE). The following information in this box may be repeated in other articles submitted by participants using the same GEP CURE protocol for annotating Drosophila species orthologs of Drosophila melanogaster genes in the insulin signaling pathway. * "In this GEP CURE protocol students use web-based tools to manually annotate genes in non-model *Drosophila* species based on orthology to genes in the well-annotated model organism fruitfly *Drosophila melanogaster* . The GEP uses web-based tools to allow undergraduates to participate in course-based research by generating manual annotations of genes in non-model species (Rele et al., 2023) . Computational-based gene predictions in any organism are often improved by careful manual annotation and curation, allowing for more accurate analyses of gene and genome evolution (Mudge and Harrow 2016; Tello-Ruiz et al., 2019) . These models of orthologous genes across species, such as the one presented here, then provide a reliable basis for further evolutionary genomic analyses when made available to the scientific community.” (Myers et al., 2024) . “The particular gene ortholog described here was characterized as part of a developing dataset to study the evolution of the Insulin/insulin-like growth factor signaling pathway (IIS) across the genus *Drosophila* . The Insulin/insulin-like growth factor signaling pathway (IIS) is a highly conserved signaling pathway in animals and is central to mediating organismal responses to nutrients (Hietakangas and Cohen 2009; Grewal 2009) .” (Myers et al., 2024) .

The model presented here is the ortholog of *ImpL2* in the DsimGB2 assembly of *D. simulans* ( *Drosophila* 12 Genomes Consortium et al., 2007; GCA_000754195.3 ) and corresponds to the *Gnomon Peptide ID ( XP_002083603.1 )* predicted model in *D. simulans ( LOC6736747 ).* This gene model is based on RNA-seq data from *D. simulans* (Gravely et al., 2011; SRP006203 ) and the *ImpL2* in *D. melanogaster* from *FB2022_02* (Larkin et al., 2021; Gramates et al., 2022; Jenkins et al., 2022; *GCA_000001215.4* ).

*Ecdysone-inducible gene L2* was originally known as ***I*** *nducible* ***m*** *embrane-bound* ***p*** *olysomal transcript* ***2*** (Natzle et al., 1986) , and is also known as *Imaginal morphogenesis protein-Late 2, ImpL2* , *Imp-L2* , or *ImpL-2* (FBgn0001257). ImpL2 is a putative insulin-binding protein, and is thought to antagonize insulin pathway activity (Honegger et al., 2008; Alic et al., 2011) . *ImpL2* function is essential in the development of the *Drosophila* nervous system during early embryogenesis (Garbe et al., 1993; Bader et al., 2013) . *Impl2* is the sole fly homolog of mammalian IGFBPs [insulin-like growth factor binding proteins]. In fly tumor models, ImpL2 acts as a secreted wasting factor that contributes to loss of tissue mass by antagonizing system-wide IGF1 signaling (Figueroa-Clarevega and Bilder 2015; Kwon et al., 2015) . It was recently demonstrated that the growth-decreasing effects of ImpL2 in model *Drosophila* midgut tumors are diminished by upregulation of Wingless (Wg) signaling, although whether this is a direct mechanism remains to be established (Lee et al., 2021) .

*D. simulans (* NCBI:txid7240) is part of the *melanogaster* species group within the subgenus *Sophophora* of the genus *Drosophila* (Sturtevant 1939; Bock and Wheeler 1972) *.* It was first described by Sturtevant (1919). *D. simulans* is a sibling species to *D. melanogaster* , thus extensively studied in the context of speciation genetics and evolutionary ecology (Powell 1997) . Historically, *D. simulans* was a tropical species native to sub-Saharan Africa (Lemeunier et al., 1986) where figs served as a primary host (Lachaise and Tsacas 1983) . However, *D. simulans's* range has expanded worldwide within the last century as a human commensal using a broad range of rotting fruits as breeding sites (https://www.taxodros.uzh.ch, accessed 1 Feb 2023).

***Synteny***

*ImpL2* occurs on chromosome 3L in *D. melanogaster. CG46460* is a dicistronic transcript that is nested within a UTR CDS of *ImpL2 .* *ImpL2* is flanked upstream by *pav* and *CG14997* and downstream by *CG43367* and *CG18675 .* C *pr64Ad* , C *pr64Ac* , C *pr64Ab* , C *pr64Aa* , and *CG15005* are nested within *CG43367 . tipE , VhaM9.7-c , Teh3 , Teh2 , Teh4* are nested within *CG18675 .* We determined that the putative ortholog of *ImpL2* is found on scaffold CM002912.1 in *D. simulans* ( GCA_000754195.3 ) with LOC6736747 ( XP_002083603.1 ) (via *tblastn* search with an e-value of 0.0 and percent identity of 99.25%). LOC120284786 ( XP_039149640.1 ), which corresponds to *CG46460* in *D. melanogaster* with an e-value of 6e-116 and a percent identity of 100%, as determined by *blastp,* is nested within the *ImpL2* ortholog in *D. simulans* . It is flanked upstream by LOC6736750 ( XP_016030374.1 ) and LOC6736749 ( XP_002083605.1 ) which correspond to *pav* and *CG14997 ,* with e-values of 0.0 and 0.0, respectively, and percent identities of 99.55% and 97.81%, respectively, as determined by *blastp.* It is flanked downstream by LOC6736738 ( XP_016030369.1 ) and LOC6736732 ( XP_002083588.1 ) *,* which correspond to *CG43367* and *CG18675* in *D. melanogaster* with e-values of 0.0 and 0.0, respectively, and percent identities of 99.23% and 96.85%, respectively, as determined by *blastp* ( Figure 1A -i, Altschul et al., 1990). LOC6736744 ( XP_016030372.1 ), LOC6736743 ( XP_002083599.1 ), LOC6736742 ( XP_002083598.1 ), LOC6736741 ( XP_002083597.1 ), and LOC6736739 ( XP_039149637.1 ), which correspond to C *pr64Ad* , C *pr64Ac* , C *pr64Ab* , C *pr64Aa* , and *CG15005* in *D. melanogaster* with e-values of 2e-65, 5e-69, 5e-54, 4e-48, and 0.0, respectively, and percent identities of 99.02%, 99.47%, 98.02%, 100%, and 96.94%, respectively, as determined by *blastp,* are nested within the *CG43367* ortholog found at LOC6736738 ( XP_016030369.1 ; Figure 1A -ii, Altschul et al., 1990) *.* LOC6736737 ( XP_016030363.1 ), LOC27206252 ( XP_016030361.1 ), LOC6736735 ( XP_016030360.1 ), LOC6736734 ( XP_002083590.1 ), and LOC6736733 ( XP_039149638.1 ), which correspond to *tipE , VhaM9.7-c , Teh3 , Teh2 , Teh4* in *D. melanogaster* with e-values of 0.0, 3e-45, 0.0, 0.0, and 0.0, respectively, and percent identities of 99.12%, 96.43%, 100%, 99.65%, and 98.66%, respectively, as determined by *blastp,* are nested within the *CG18675* ortholog found at LOC6736732 ( XP_002083588.1 ; 1A-iii, Altschul et al., 1990). We believe this is the correct ortholog assignment for *ImpL2* in *D. simulans* because local synteny is completely conserved and because all of the *blastp* results used to determine the orthology of neighboring genes are very high-quality matches.

***Protein Model***

*ImpL2* in *D. simulans* has three unique protein coding isoforms ( Figure 1B ). ImpL2-PA and ImpL2-PC contain three CDSs, the first of which is different and the other two are the same. mRNAs *ImpL2-RB* and *ImpL2-RD* , which differ in their 3' UTRs, contain the same two CDSs. Relative to the ortholog in *D. melanogaster,* all isoforms are present in *D. simulans* and have the same respective number of CDSs *.* The sequence of ImpL2-PC in *D. simulans* has 99.3% identity similarity with ImpL2-PC in *D. melanogaster* as determined by *blastp* ( Figure 1C ). Note that the annotation for the first CDS of *ImpL2-PA (Flybase ID: 1_294_0) d* oes not align with the Spaln of *D. melanogaster* Proteins track prediction for the first CDS of the isoform (Flybase IDs from FB2022_03). The placement of this CDS in our model was determined based multiple lines of evidence including the BLAT alignments of NCBI RefSeq Genes, RNA-Seq from adult male and females, Transcripts and Coding Regions Predicted by TransDecoder, and Splice Junctions Predicted by regtools using *D. simulans* RNA-Seq, all of which support the CDS we selected. Additionally, the peptide sequence of this first CDS in *D. melanogaster* is MQ, which is identical to the sequence of the chosen orthologous placement in *D. simulans* , whereas the Spaln of *D. melanogaster* Proteins track prediction indicates a CDS with peptide sequence MS. This combined data provides evidence to support the determined location of the first CDS of ImpL2-PA (Flybase ID: 1_294_0) in *D. simulans* . The coordinates of the curated gene models can be found in NCBI at GenBank/BankIt using the accessions BK063005 , BK063006 , BK063007 , and BK063008 . These data are also available in Extended Data files below, which are archived in CaltechData.

## Methods

Detailed methods including algorithms, database versions, and citations for the complete annotation process can be found in Rele et al. (2023). Briefly, students use the GEP instance of the UCSC Genome Browser v.435 ( https://gander.wustl.edu ; Kent WJ et al., 2002; Navarro Gonzalez et al., 2021) to examine the genomic neighborhood of their reference IIS gene in the *D. melanogaster* genome assembly (Aug. 2014; BDGP Release 6 + ISO1 MT/dm6). Students then retrieve the protein sequence for the *D. melanogaster* target gene for a given isoform and run it using *tblastn* against their target *Drosophila* species genome assembly ( *Drosophila simulans* ( GCA_000754195.3 - Graveley et al., 2010)) on the NCBI BLAST server ( https://blast.ncbi.nlm.nih.gov/Blast.cgi , Altschul et al., 1990) to identify potential orthologs. To validate the potential ortholog, students compare the local genomic neighborhood of their potential ortholog with the genomic neighborhood of their reference gene in *D. melanogaster* . This local synteny analysis includes at minimum the two upstream and downstream genes relative to their putative ortholog. They also explore other sets of genomic evidence using multiple alignment tracks in the Genome Browser, including BLAT alignments of RefSeq Genes, Spaln alignment of D. melanogaster proteins, multiple gene prediction tracks (e.g., GeMoMa, Geneid, Augustus), and modENCODE RNA-Seq from the target species. Genomic structure information (e.g., CDSs, CDS number and boundaries, number of isoforms) for the *D. melanogaster* reference gene is retrieved through the Gene Record Finder ( https://gander.wustl.edu/~wilson/dmelgenerecord/index.html ; Rele et al *.,* 2023). Approximate splice sites within the target gene are determined using *tblastn* using the CDSs from the *D. melanogaste* r reference gene. Coordinates of CDSs are then refined by examining aligned modENCODE RNA-Seq data, and by applying paradigms of molecular biology such as identifying canonical splice site sequences and ensuring the maintenance of an open reading frame across hypothesized splice sites. Students then confirm the biological validity of their target gene model using the Gene Model Checker ( https://gander.wustl.edu/~wilson/dmelgenerecord/index.html ; Rele et al., 2023), which compares the structure and translated sequence from their hypothesized target gene model against the *D. melanogaster* reference gene model. At least two independent models for each gene are generated by students under mentorship of their faculty course instructors. These models are then reconciled by a third independent researcher mentored by the project leaders to produce a final model like the one presented here. Note: comparison of 5' and 3' UTR sequence information is not included in this GEP CURE protocol.

## Extended Data

Description: Model Data (FNA, FAA, GTF). Resource Type: Model. DOI: 10.22002/ffnsa-1r156
